# Altered Vascular Adaptations to Pregnancy in a Rat Model of Advanced Maternal Age

**DOI:** 10.3389/fphys.2021.718568

**Published:** 2021-07-28

**Authors:** Mazhar Pasha, Amy L. Wooldridge, Raven Kirschenman, Floor Spaans, Sandra T. Davidge, Christy-Lynn M. Cooke

**Affiliations:** ^1^Department of Physiology, University of Alberta, Edmonton, AB, Canada; ^2^Department of Obstetrics and Gynecology, University of Alberta, Edmonton, AB, Canada; ^3^Women and Children’s Health Research Institute, University of Alberta, Edmonton, AB, Canada

**Keywords:** advanced maternal age, mesenteric artery, wire myography, endothelium-dependent relaxation, pregnancy adaptations

## Abstract

Advanced maternal age (≥35 years old) increases the risk of pregnancy complications such as preeclampsia and fetal growth restriction. We previously demonstrated vascular dysfunction and abnormal pregnancy outcomes in a rat model of advanced maternal age. However, vascular adaptations to pregnancy in aging were not studied. We hypothesize that advanced maternal age is associated with a more vasoconstrictive phenotype due to reduced nitric oxide (NO) and increased activity of matrix metalloproteinases (MMPs), contributing to impaired vascular adaptations to pregnancy. A rat model of advanced maternal age was used: young (4 months) and aged (9.5 months; ∼35 years in humans) non-pregnant and pregnant rats. On gestational day 20 (term = 22 days; non-pregnant rats were aged-matched), blood pressure and heart rate were measured (tail cuff plethysmography) and vascular function was assessed in mesenteric arteries (wire myography). Endothelium-dependent relaxation to methylcholine (MCh) was assessed in the presence/absence of nitric oxide synthase inhibitor (L-NAME), or inhibitors of endothelium-dependent hyperpolarization (EDH; apamin and TRAM-34). Vasoconstriction responses to big endothelin-1 (bigET-1), in the presence/absence of MMPs-inhibitor (GM6001) or endothelin converting enzyme (ECE-1) inhibitor (CGS35066), in addition, ET-1 responsiveness, were measured. Blood pressure was elevated only in aged non-pregnant rats (*p* < 0.001) compared to all other groups. MCh responses were not different, however, L-NAME decreased maximum vasodilation in young (*p* < 0.01) and aged pregnant rats (*p* < 0.001), and decreased MCh sensitivity in young non-pregnant rats (*p* < 0.01), without effects in aged non-pregnant rats. EDH contribution to relaxation was similar in young non-pregnant, and aged non-pregnant and pregnant rats, while EDH-mediated relaxation was absent in young pregnant rats (*p* < 0.001). BigET-1 responses were enhanced in aged non-pregnant (*p* < 0.01) and pregnant rats (*p* < 0.05). No significant changes in bigET-1 conversion occurred in the presence of MMP-inhibitor, whereas ECE-1 inhibition reduced bigET-1 constriction in aged rats (*p* < 0.01). No differences in ET-1 sensitivity were observed. In conclusion, contrary to our hypothesis, reduced blood pressure, and an increased EDH-dependent contribution to vasodilation suggest a compensatory mechanism that may reflect beneficial adaptations in these aged rats that were able to maintain pregnancy. These data increase our understanding of how the vascular adaptive pathways in pregnancy compensate for advanced maternal age.

## Introduction

Advanced maternal age is defined as maternal age ≥35 years at the time of delivery. The age at which women deliver their first child has been increasing steadily in North America, accounting for 14–18% of total live births from women aged 35 years (Garner and Bushnik, 2008; Martin et al., 2009; Canada, 2012). Studies have shown advanced maternal age increases the risk of pregnancy-related complications such as fetal growth restriction (FGR), preeclampsia, gestational diabetes, hypertension, small for gestational age infants, preterm birth, and stillbirth (Yoon et al., 1996; Salihu et al., 2008; Lamminpaa et al., 2012; Ludford et al., 2012; Kenny et al., 2013; Khalil et al., 2013). A large contemporary cohort study in the United Kingdom revealed an 18.2% prevalence of advanced maternal age (Kenny et al., 2013). In addition, a population based-cohort study in South Australia showed that women of advanced age were prone to have adverse maternal perinatal outcomes (Ludford et al., 2012). These studies clearly delineate that advanced maternal age is becoming a global health challenge and women of advanced age represent a very important and yet understudied population of pregnant women.

Healthy maternal vascular adaptations to pregnancy play an essential role in normal fetal growth and development. To achieve this, several hemodynamic changes occur, such as increased cardiac output, increased heart rate, and increased blood volume, while there is a decrease in blood pressure in early- to mid-gestation due to a reduced systemic vascular resistance during pregnancy (Wang et al., 2015; Donato et al., 2018; Ma et al., 2020). Though the mechanisms of this reduced peripheral resistance are not completely understood, multiple studies have shown that during pregnancy the bioavailability of nitric oxide (NO), a potent endothelium-derived vasodilator, is increased (Dorup et al., 1999; Valdes et al., 2009; Boeldt and Bird, 2017). In addition, vasodilation of small resistance arteries (mesenteric arteries) during pregnancy is also mediated by other factors, such as endothelium-derived hyperpolarization (EDH) (Gerber et al., 1998; Cooke and Davidge, 2003; Mandala et al., 2012). Though EDH is distinct from NO, it has been shown that if NO-mediated relaxation is decreased, EDH may compensate for endothelium-dependent relaxation (Takaki et al., 2008). Moreover, studies have shown upregulation of EDH in normal pregnancy (in resistance-sized myometrial arteries and small subcutaneous arteries), that may be absent in pregnancy-related complication such as preeclampsia (Kenny et al., 2002; Luksha et al., 2004).

In women of advanced maternal age, normal pregnancy-induced vascular adaptations may be impaired, leading to poor pregnancy outcomes and altered vascular function. We have previously demonstrated abnormal pregnancies in aged rats, with reduced capacity to sustain a pregnancy, adverse pregnancy outcomes, and altered vascular function with increased active myogenic responses in both uterine and mesenteric arteries from aged pregnant rats (Care et al., 2015). The maternal uterine arteries undergo significant vascular remodeling during gestation to support the developing fetus and poor vascular adaptations in the uterine vessels have been associated with common pregnancy related complications, including intrauterine growth restriction and preeclampsia (Magness et al., 1996, 1997; Sladek et al., 1997; Osol and Mandala, 2009; Osol and Moore, 2014). Similarly, an impaired adaptation response to pregnancy (hormonal, immunological, and cardiovascular dysfunction) and postnatal growth restriction was shown using a vervet monkey model of advanced maternal age (Plant et al., 2020). Further, in mice, impaired utero-placental vascular function and placental dysfunction were proposed as potential mechanisms for the increased susceptibility to FGR and stillbirth associated with advanced maternal age (Lean et al., 2017; Napso et al., 2019). Overall, these studies suggest that pregnancy-induced vascular adaptations may be suboptimal in advanced maternal age pregnancies, eventually increasing the risk of pregnancy-related complications. However, despite the fact that pregnancies at advanced maternal age are considered high-risk clinically, very little research has focused on understanding the systemic vascular pathways that may be altered in advanced maternal age, leading to abnormal pregnancy adaptations and poor pregnancy outcomes for these women and their children (Ludford et al., 2012; Kenny et al., 2013; Care et al., 2015; Plant et al., 2020).

Aging itself is an independent risk factor for cardiovascular disease (CVD) (Shirasaki et al., 1986; Brandes et al., 2005; Kruger-Genge et al., 2019). The most noticeable characteristics are altered structural and functional properties of the endothelium and vascular smooth muscle cells leading to vascular remodeling, increased vascular stiffness, and endothelial dysfunction (Harvey et al., 2015; Xu et al., 2017; Dong et al., 2019). To maintain vascular homeostasis, there is a fine balance between vasodilator (NO) and vasoconstrictor agents (e.g., endothelin-1 (ET-1)) (Cardillo et al., 2000; Versari et al., 2009); and although the pathophysiological mechanisms leading to endothelial dysfunction are likely multifactorial, a hallmark of endothelial dysfunction in the aging vasculature was shown to be reduced bioavailability of NO and enhanced reactivity of ET-1 (Boss and Seegmiller, 1981; Toda, 2012; Xu et al., 2017; Dong et al., 2019). ET-1 is a potent vasoconstrictor polypeptide and has the capacity to induce vascular remodeling, thus ET-1 signaling is believed to be one of the important contributors to the progression of vascular dysfunction in aging and cardiovascular disease (Bohm and Pernow, 2007; Ergul, 2011). In addition, studies have also shown a significant increase in circulating levels of ET-1 in women with pregnancy-related complications (e.g., preeclampsia and FGR) compared with normal pregnant women (Bernardi et al., 2008; Dieber-Rotheneder et al., 2012). The vasoactive peptide ET-1 is synthesized from its precursor big endothelin-1 (bigET-1), and subsequently cleaved by several enzymes, such as matrix metalloproteases (MMPs) (Abdalvand et al., 2013), endothelin converting enzymes (ECE-1) (Parnot et al., 1997; Kuruppu and Smith, 2012), chymase (Tirapelli et al., 2006), and neutral endopeptidases (Ferro et al., 1998). Studies have demonstrated that MMPs and ECE-1 play essential roles in the processing of bigET-1 to ET-1 under various pathological conditions such as aging (Lekontseva et al., 2010), preeclampsia (Ajne et al., 2003; Abdalvand et al., 2013), and hypoxia (He et al., 2007). In addition, MMPs also maintain the stability of the extracellular matrix by degrading collagen, elastin, and other extracellular proteins as part of the normal physiological and pathological processes (Chen et al., 2013). Although MMPs play a significant role during healthy pregnancies (e.g., in trophoblast invasion of the uterus) (Cohen et al., 2012), several studies showed that with advancing age, dysregulation of MMPs activity may contribute to endothelial dysfunction (Jackson and McArdle, 2011; Donato et al., 2018; Liguori et al., 2018). However, the role of MMPs in vascular adaptations to pregnancy with advanced maternal age is not known.

Both human and animal studies have shown the importance of systemic vascular adaptations to pregnancy (Veerareddy et al., 2002; Cooke and Davidge, 2003; Barry and Anthony, 2008; Amburgey et al., 2010; Kahveci et al., 2018; Albrecht et al., 2021). However, the impact of age-related vascular impairments during pregnancy, such as altered endothelium-dependent vasodilation (reduced NO bioavailability) and increased endothelium-derived contracting factors (e.g., ET-1) due to their processing enzymes (such as MMPs) remains elusive. Thus, the focus of the current study is to increase our understanding of how these vascular pathways impact the systemic vascular adaptations to pregnancy in an established rat model of advanced maternal age. We used mesenteric arteries as they are a systemic, resistance-sized artery that plays an important role in the control of blood pressure (Christensen and Mulvany, 1993). We hypothesize that maternal aging impairs systemic vascular adaptations to pregnancy, due to a more vasoconstrictive phenotype associated with reduced NO contribution and increased activity of MMPs in the aging vasculature.

## Materials and Methods

### Ethical Approval

All experimental procedures received prior approval by the University of Alberta Health Sciences Animal Policy and Welfare Committee, in accordance with the guidelines of the Canadian Council on Animal Care and the Guide for the Care and Use of Laboratory Animals published by the US National Institutes of Health (AUP #3693).

### Animal Model and Experimental Design

Female and male (for breeding) Sprague Dawley (SD) rats were purchased at 3 months of age from Charles River Canada (Saint-Constant, QC, Canada). The rats were housed at an ambient temperature of 22 ± 1°C and a 10:14 h light: dark cycle. All rats were allowed at least 1 week of acclimatization after arrival. We used a rat model of advanced maternal age previously established and characterized in our laboratory (Care et al., 2015; Cooke et al., 2018; Shah et al., 2018). Briefly, aged non-pregnant and pregnant rats were 9.5–10 months of age, which corresponds to ≈35 years of age in humans (considering milestones: weaning, skeletal maturity, sexual maturity, and reproductive senescence) (Quinn, 2005); whereas young non-pregnant and pregnant rats were 3–4 months of age (equivalent to early reproductive maturity in humans) (Quinn, 2005; Cooke et al., 2018). Young rats had *ad libitum* access to standard rat chow, while aging rats were fed a restricted diet of six pellets per day for caloric and nutrient intake based on National Research Council recommendations, to prevent age-related obesity as a confounding factor (National Research Council (US) Subcommittee on Laboratory Animal Nutrition, 1995). Young and aged female rats were mated with young male rats (aged 3–5 months), and once pregnancy was confirmed by the presence of sperm in a vaginal smear (designated as gestational day GD 0), all rats were fed an *ad libitum* standard chow diet (Care et al., 2015; Cooke et al., 2018). On GD20 (term = 22 days), blood pressure was assessed, rats were anesthetized using isoflurane and euthanized by exsanguination via cardiac puncture. The mesenteric arcade was immediately excised and placed in ice-cold HEPES-buffered physiological saline solution [PSS; composition (in mM): 10 HEPES, 5.5 glucose, 1.56 CaCl_2_, 4.7 KCl, 142 NaCl, 1.17 MgSO_4_, 1.18 KH_2_PO_4_, and pH 7.4], after which the mesenteric arteries were isolated for *ex vivo* vascular function and Western blot analysis.

### Blood Pressure and Heart Rate Measurements

Blood pressure and heart rate were measured before euthanasia on GD20 by tail-cuff plethysmography (CODA High Throughput System, Kent Scientific, Torrington, CT, United States). All rats were subjected to a 1-week training period (i.e., to get used to the nose cone holders and occlusion tail cuff) for acclimatization to the system before pregnancy. To obtain the cardiovascular parameters, rats were placed in the nose cone holders for 20 min to warm up (tail skin surface temperature ∼30°C) after which at least 10 consecutive blood pressure measurements (systolic, diastolic, and mean arterial pressure (MAP)) and heart rate measurements were recorded and averaged for each rat (Cooke et al., 2018).

### Mesenteric Artery Vascular Function by Wire Myography

Vascular function was assessed *ex vivo* in isolated systemic mesenteric (small resistance) arteries using wire myography. Second-order mesenteric arteries (150–250 μm) were isolated and cleaned from the perivascular fat in ice-cold PSS and cut into 2 mm pieces. The 2 mm segments of mesenteric artery were mounted onto a wire myograph system (620M DMT, Copenhagen, Denmark) using 40 μm tungsten wires. Isomeric tension of the vessels was recorded using LabChart software (AD Instruments; Colorado Springs, United States). All vessels were normalized through a series of stepwise increases in diameter set their diameter to their optimal resting tension: 0.8 × IC100; 13.3 kPa (the internal circumference equivalent to a transmural pressure of 100 mmHg) (Davidge et al., 1993). After normalization the vessels were exposed to the first wake up dose of phenylephrine (10 μM; Sigma-Aldrich, St. Louis, MO, United States) for 5 min. After washing with PSS thrice and 10 min rest, the vessels were exposed to a second wake up dose of phenylephrine followed by a single dose of methylcholine (MCh; 3 μM; Sigma-Aldrich) to confirm endothelial cell function. After 30 min of rest, vascular responses to MCh were assessed using a cumulative concentration response curve (CCRCs; 0.003 to 3 M MCh) after pre-constriction with the EC_80_ concentration (3 μM; the mean effective concentration that produces 80% of the maximal response) of phenylephrine. To assess relevant pathways involved in endothelium-dependent vasodilation, the CCRCs to MCh were performed in the absence or presence (30 min pre-incubation prior to phenylephrine EC_80_ dose) of the following specific inhibitors: NO synthase (NOS) was inhibited with the pan NOS inhibitor N(G)-nitro-L-arginine methyl ester hydrochloride (L-NAME; 100 μM; Sigma-Aldrich) and EDH-induced vasodilation was inhibited with a combination of apamin (100 nM) (Sigma-Aldrich) which blocks small-conductance Ca^2+^-activated potassium channels (SK), and 1-(2-chlorophenyl)diphenylmethyl-1H-pyrazole (TRAM-34; 10 μM; Sigma-Aldrich), an intermediate-conductance Ca^2+^-activated potassium channel (IK) inhibitor. In addition, contribution of the myoendothelial gap junctions (MEGJs) to EDH responses was assessed with 18 α-glycyrrhetinic acid (3 μM), which inhibits MEGJs. Simultaneously, a CCRC to the NO donor sodium nitroprusside (0.003 to 2 M SNP; Sigma-Aldrich) was conducted in one of the baths to assess endothelium-independent relaxation. Simultaneously, in separate myography baths, vasoconstriction responses to big endothelin-1 (bigET-1) and endothelin-1 (ET-1) were assessed, using CCRCs to ET-1 (1–200 nM; Sigma-Aldrich) or bigET-1 (3–310 nM; AnaSpec, Fremont, United States) to assess the capacity of the vessel to convert bigET-1 to ET-1. Constriction responses to bigET-1 were assessed in the absence or presence of inhibitors of the bigET-1 converting enzymes: GM6001 (30 μM; Calbiochem), a broad spectrum MMPs inhibitor; CGS35066 (25 μM; Tocris Bioscience, Toronto, Canada), a selective endothelin converting enzyme (ECE-1) inhibitor; chymostatin (100 μM; Sigma-Aldrich), a chymase inhibitor; and DL-Thiorphan (25 μM; Calbiochem), a selective inhibitor of neutral endopeptidase. Finally, all vessels were washed 4 times with PSS, allowed to rest for 15 min, and each bath was exposed to a 124 mmol/L potassium chloride solution (high KCl buffer; containing in mM: 10 HEPES, 24 NaCl, 124 KCl, 2.4 MgSO_4_, 4.9 CaCl_2_, 1.18 KH_2_PO_4_, and 5.5 glucose; pH 7.4) to assess maximum non-receptor-mediated smooth muscle vasoconstriction responses. All data [maximum relaxation responses to MCh (E_max_), the negative log of the mean effective concentration that produces 50% of the maximal response (pEC_50_) as a measure of sensitivity to the vasodilator/vasoconstrictor, and the area under the cure (AUC)], were analyzed using GraphPad Prism 9 (GraphPad Software, San Diego, United States).

### Expression of eNOS and ECE-1 Using Western Blot Analysis

Mesenteric arteries were isolated, cleaned from perivascular fat and homogenized using lysis buffer [concentration in mM: 20 Tris (pH 7.4), 10 sodium pyrophosphates tetrabasic, 100 sodium, 5 EDTA, and 9 fluoride with 1% Nonidet P-40] containing protease inhibitor cocktail (Thermo Fisher Scientific), phosphatase inhibitor (2 mM sodium orthovanadate, Sigma) and 1 mM Phenylmethylsulfonyl fluoride (PMSF; Fluka BioChemika). Total protein concentration of the samples was determined using bicinchoninic acid assay (Pierce, Rockford, IL, United States). Mesenteric artery tissue homogenates (50 μg of protein) were loaded and separated on 7% SDS-polyacrylamide gels for assessment of endothelial nitric oxide synthase (eNOS) and ECE-1 expression. Gels were then transferred to a nitrocellulose membrane (100V, 1.5 h; 0.2 μm, Bio-Rad), after which the membrane was stained with LI-COR Revert 700 Total Protein Stain and imaged using the LI-COR Odyssey system. Following reversal of the total protein staining, membranes were incubated with BlockOut^®^-Universal Blocking Buffer (Rockland, PA, United States) for 1 h at room temperature to prevent non-specific binding of the antibodies. Membranes were then incubated overnight at 4°C with primary antibodies for phospho-eNOS (Ser1177) (1:500 rabbit polyclonal, Cell signaling technology), total eNOS (1:1000 mouse monoclonal, Thermo Fisher Scientific) and ECE-1 (1:500 mouse monoclonal, Santa Cruz Biotechnology) in phosphate buffered saline with Tween [PBST; NaCl: 137 mM, KCl: 2.7 mM, Na_2_HPO_4_: 10 mM, KH_2_PO_4_: 1.8 mM, and Tween^®^ 20 detergent: 0.1% (w/v); Thermo Fisher Scientific]. The next day, membranes were incubated with the appropriate secondary antibody: IRDye donkey anti-rabbit IgG (for phospho-eNOS) and IRDye donkey anti-mouse IgM (for total eNOS and ECE-1) at 1:10,000 in PBST buffer (LI-COR Biosciences, Lincoln, NE, United States). Finally, blots were visualized with a LI-COR Odyssey Bioimager (LI-COR Biosciences) and quantified using Image Studio Lite software (LI-COR Biosciences). All data are expressed as percent change compared to the corresponding control (young non-pregnant rats) for each of the separate blots.

### Statistical Analyses

Data were plotted using GraphPad Prism 9 (GraphPad Software, San Diego, United States) and presented as mean ± SEM, and were analyzed using a two-way ANOVA with Sidak’s *post hoc* test for multiple comparisons. *P*-values < 0.05 were considered statistically significant.

## Results

### Blood Pressure and Heart Rate Assessment in Young Compared to Aged Non-pregnant and Pregnant Rats

**FIGURE 1:**
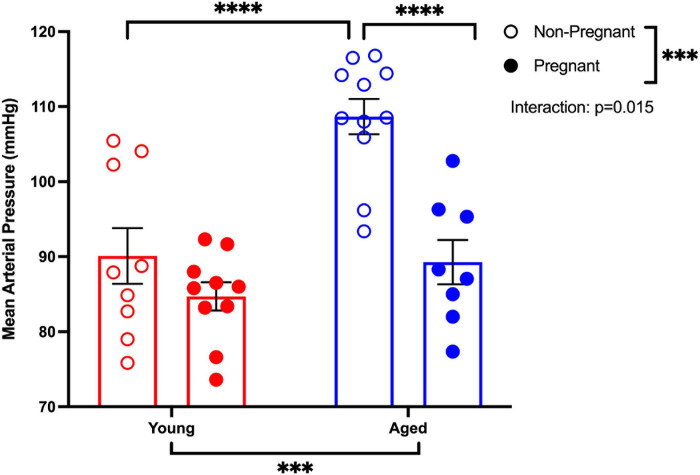
Pregnancy reduced the mean arterial pressure in aged pregnant rats. Mean arterial blood pressure recordings (MAP) of young (3–4 months; in red) and aged (9–9.5 months; in blue) pregnant (on gestational day 20; closed circles) and non-pregnant (age-matched; open circles) rats. Data presented as mean ± SEM; analyzed by two-way ANOVA with Sidak’s multiple comparisons *post hoc* test; ^∗∗∗^*p* < 0.001, ^*⁣*⁣**^p < 0.0001; *n* = 9–10/group.

MAP was increased in aged non-pregnant rats compared to aged pregnant, young non-pregnant, and pregnant rats (Figure 1). Pregnancy reduced the MAP in aged pregnant rats compared to aged non-pregnant rats to pressures similar to those of the young pregnant rats. However, no changes in MAP were observed between young non-pregnant and pregnant rats (Figure 1). A similar pattern of changes was observed with the systolic and diastolic blood pressures. Moreover, heart rates tended to be higher in young pregnant rats compare to young non-pregnant rats, while this effect was not observed between the aged non-pregnant and pregnant rats (Supplementary Table 1).

### Vasodilation Pathways

#### Endothelium-Dependent Vasodilation and NO Contribution to Vasodilation

**FIGURE 2:**
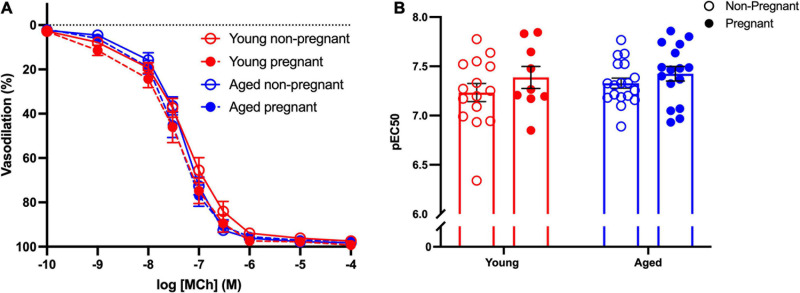
No differences in the endothelium-dependent vasodilation in young and aged non-pregnant and pregnant rats. **(A)** Endothelium-dependent vasodilation responses to increasing doses of methylcholine (MCh) in mesenteric arteries of young (3–4 months; in red) and aged (9–9.5 months; in blue) pregnant (on gestational day 20; closed circles) and non-pregnant (age-matched; open circles) rats. **(B)** Data summary of sensitivity to MCh (pEC_50_). Data are presented as mean ± SEM; analyzed by two-way ANOVA with Sidak’s multiple comparisons *post hoc* test; *n* = 9–16/group.

**FIGURE 3:**
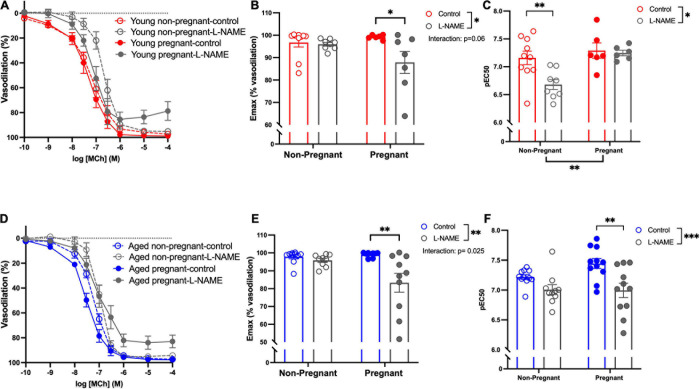
Increased nitric oxide-dependent vasodilation during pregnancy in mesenteric arteries from young and aged pregnant rats. **(A,D)** Endothelium-dependent vasodilation responses to increasing doses of methylcholine (MCh) in the presence or absence of L-NAME in mesenteric arteries of young [3–4 months; in red; **(A–C)**] and aged [9–9.5 months; in blue; **(D–F)**] pregnant (on gestational day 20; closed circles) and non-pregnant (age-matched; open circles) rats. **(B,E)** Data summaries of the maximal vasodilation responses to MCh (E_max_). **(C,F)** Data summaries of the sensitivity to MCh (pEC_50_). Data are presented as mean ± SEM; analyzed by two-way ANOVA with Sidak’s multiple comparisons *post hoc* test; ^∗^*p* < 0.05, ^∗∗^*p* < 0.01; ^∗∗∗^*p* < 0.001; *n* = 6–11/group.

**FIGURE 4:**
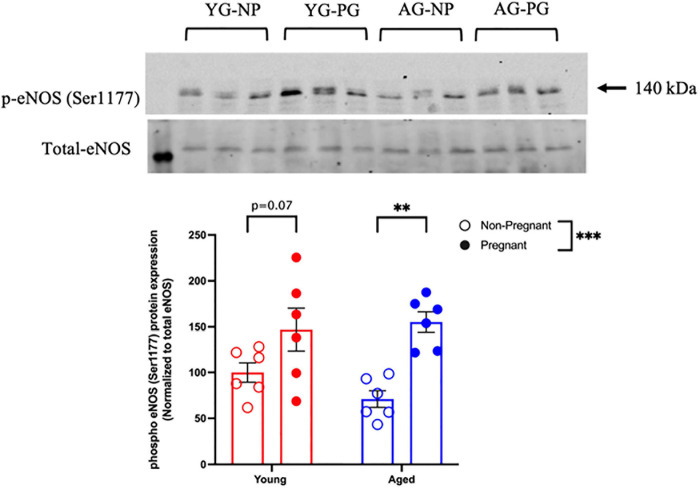
Increased phosphorylation of eNOS protein in young and aged pregnant rats. Western blot analysis of phospho-eNOS (Ser1177) normalized to total eNOS in mesenteric arteries of young (3–4 months; in red) and aged (9–9.5 months; in blue) pregnant (on gestational day 20; closed circles) and non-pregnant (age-matched; open circles) rats. Data are presented as mean ± SEM and expressed as percentage of control (i.e., the mean of the non-pregnant young group); analyzed by two-way ANOVA with Sidak’s multiple comparisons *post hoc* test; ^∗∗^*p* < 0.001; ^∗∗∗^*p* < 0.001; *n* = 6/group. YG-NP, young non-pregnant; YG-PG, young pregnant; AG-NP, aged non-pregnant; AG-PG, aged pregnant.

Methacholine (MCh)-induced vasodilation responses were not different in mesenteric arteries from young or aged, non-pregnant or pregnant rats (Figures 2A,B and Supplementary Table 2). However, pre-incubation with L-NAME (to assess NO contribution) decreased the E_max_ to MCh in arteries from both young pregnant and aged pregnant rats, while there was no effect of L-NAME on MCh E_max_ in young and aged non-pregnant rats (Figures 3A,B,D,E). Moreover, L-NAME decreased the sensitivity to MCh (pEC_50_) only in young non-pregnant (Figure 3C) and aged pregnant rats (Figure 3F). Similarly, a decrease in the AUC was observed after pre-incubation with L-NAME in young non-pregnant, pregnant, and aged pregnant rats, but not in aged non-pregnant rats (Supplementary Table 3). We used Western blot to quantify eNOS protein levels/phosphorylation (at Ser1177) mesenteric arteries. There were no significant differences (*p* = 0.07) in phosphorylation of eNOS between young non-pregnant and pregnant rats, however, phosphorylation of eNOS was significantly increased in vessels from aged pregnant rats compared to aged non-pregnant rats (Figure 4). Moreover, no changes in total eNOS protein expression were observed between the groups (Supplementary Figure 1).

#### Endothelium Derived Hyperpolarization (EDH)-Mediated Vasodilation

**FIGURE 5:**
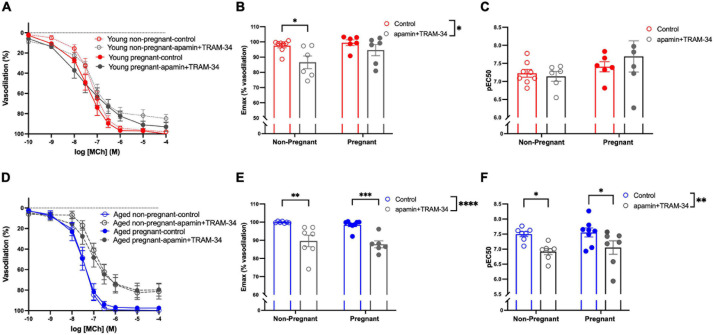
Increased endothelium-derived hyperpolarization-mediated relaxation in mesenteric arteries from aged non-pregnant and pregnant rats. **(A,D)** Endothelium-dependent vasodilation responses to increasing doses of methylcholine (MCh) in the presence or absence of apamin/TRAM-34 in mesenteric arteries of young [3–4 months; in red; **(A–C)**] and aged [9–9.5 months; in blue; **(D–F)**] pregnant (on gestational day 20; closed circles) and non-pregnant (age-matched; open circles) rats. **(B,E)** Data summaries of the maximal vasodilation responses to MCh (E_max_). **(C,F)** Data summaries of the sensitivity to MCh (pEC_50_). Data are presented as mean ± SEM; analyzed by two-way ANOVA with Sidak’s multiple comparisons *post hoc* test; ^∗^*p* < 0.05, ^∗∗^*p* < 0.01, ^∗∗∗^*p* < 0.001, ^*⁣*⁣**^p < 0.0001; *n* = 6–8/group.

Assessment of the contribution of EDH (using inhibitors apamin and TRAM-34) showed that the E_max_ and sensitivity to MCh (pEC_50_) was decreased after incubation with apamin and TRAM-34 in young non-pregnant, aged non-pregnant and aged pregnant rats while no effect of apamin and TRAM-34 was observed in young pregnant rats (Figures 5A–F). Apamin and TRAM-34 also decreased AUC in aged non-pregnant and pregnant rats (Supplementary Table 4). Inhibition of myoendothelial gap junctions (MEGJs) by 18α-glycyrrhetinic acid had no effect on MCh-induced vasodilation responses in mesenteric arteries from either young or aged, pregnant, or non-pregnant rats (Supplementary Table 5).

#### Endothelium-Independent Vasodilation

Sodium nitroprusside (SNP) dose-response curves were conducted to assess potential differences in endothelium-independent relaxation. There were no changes in the vascular responses between the young and aged, non-pregnant, and pregnant rats (Supplementary Table 6).

### Vasoconstrictor Pathways

#### Vascular Responses to BigET-1 in Mesenteric Arteries and Contribution of Converting Enzymes

**FIGURE 6:**
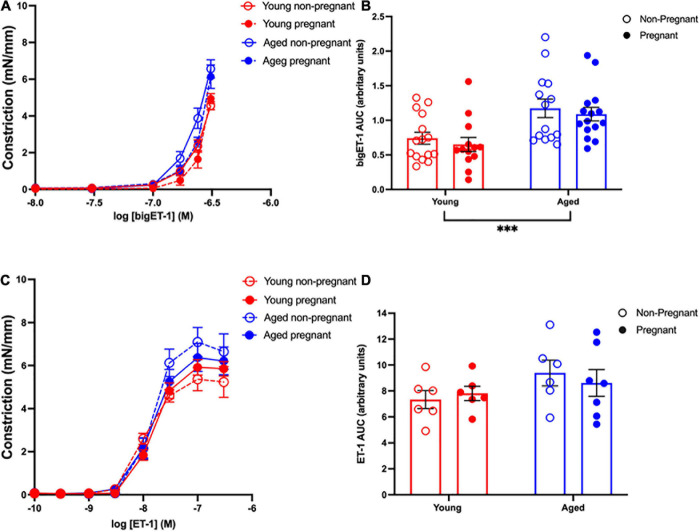
Increased vascular responses to bigET-1 in aged non-pregnant and pregnant rats, and no changes in vascular responses to ET-1. **(A,C)** Vascular contraction responses to bigET-1 and ET-1 in mesenteric arteries of young (3–4 months; in red) and aged (9–9.5 months; in blue) pregnant (on gestational day 20; closed circles) and non-pregnant (age-matched; open circles) rats. **(B,D)** Data summaries of area under the curve (AUC) of the bigET-1 **(B)** and ET-1 **(D)** responses. Data are presented as mean ± SEM; analyzed by two-way ANOVA with Sidak’s multiple comparisons *post hoc* test; ^∗∗∗^*p* < 0.001; *n* = 6–16/group.

**FIGURE 7:**
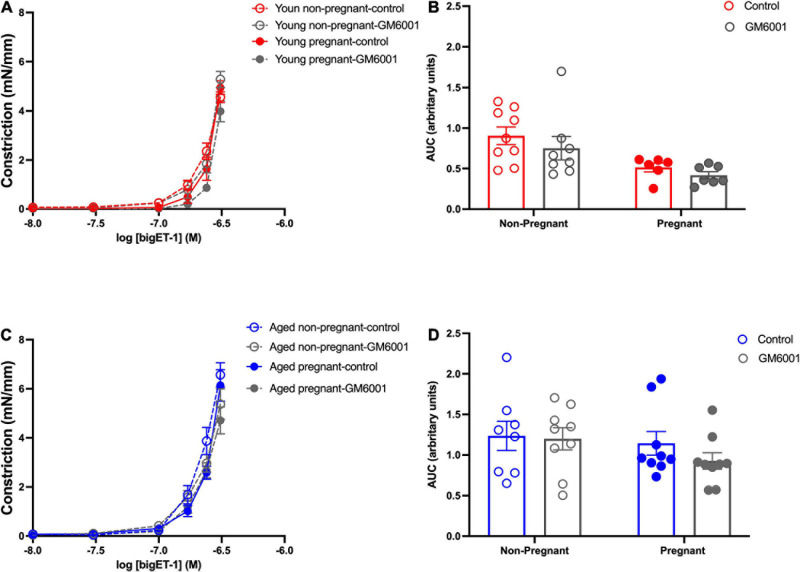
No significant contribution of matrix metalloproteases in converting to bigET-1 in young and aged non-pregnant and pregnant rats. **(A,C)** Vascular contraction response to bigET-1 in the presence or absence of MMPs inhibitor GM6001 in mesenteric arteries of young [3–4 months; in red; **(A,B)**] and aged [9–9.5 months; in blue; **(C,D)**] pregnant (on gestational day 20; closed circles) and non-pregnant (age-matched; open circles) rats. **(B,D)** Data summaries of area under the curve (AUC) of the bigET-1 responses. Data are presented as mean ± SEM; analyzed by two-way ANOVA with Sidak’s multiple comparisons *post hoc* test; *n* = 6-8/group.

**FIGURE 8:**
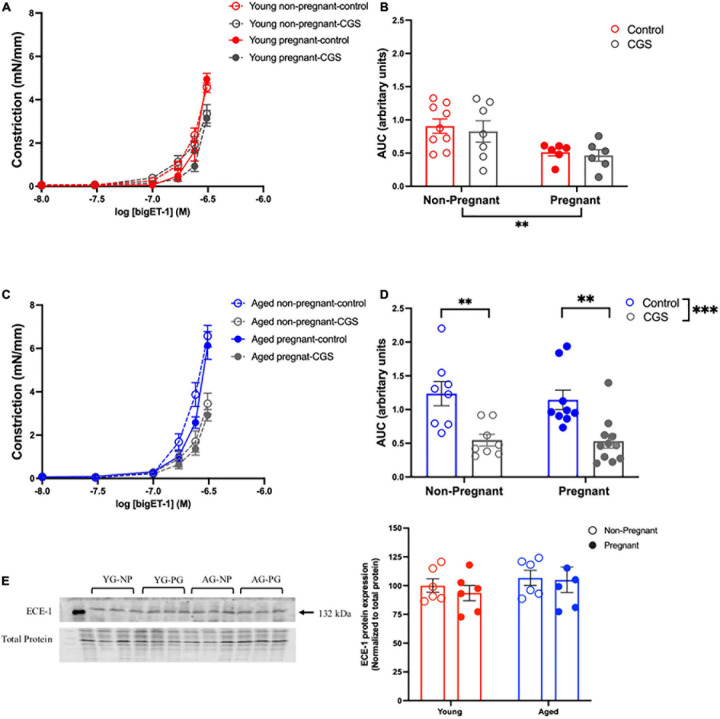
Increased contribution of endothelin converting enzyme (ECE) in converting bigET-1 in aged non-pregnant and pregnant rats. **(A,C)** Vascular contraction response to bigET-1 in the presence or absence of ECE-1 inhibitor CGS35066 (CGS) in mesenteric arteries of young [3–4 months; in red; **(A,B)**] and aged [9–9.5 months; in blue; **(C,D)**] pregnant (on gestational day 20; closed circles) and non-pregnant (age-matched; open circles) rats. **(B,D)** Data summaries of area under the curve (AUC) of the bigET-1 responses. **(E)** Western blot analysis of ECE-1 protein normalized to total protein in mesenteric arteries; the ECE-1 data is expressed as percentage of control (the mean of the non-pregnant young group). Data are presented as mean ± SEM; analyzed by two-way ANOVA with Sidak’s multiple comparisons *post hoc* test; ^∗∗^*p* < 0.001; ^∗∗∗^*p* < 0.001; *n* = 6–8/group. YG-NP, young non-pregnant; YG-PG, young pregnant; AG-NP, aged non-pregnant; AG-PG, aged pregnant.

Vasoconstriction responses to bigET-1 were enhanced in vessels from both aged non-pregnant and pregnant rats compared to the young non-pregnant and pregnant rats, however, there was no significant effect of pregnancy (Figures 6A,B and Supplementary Table 7). The enhanced constriction responses to bigET-1 in aged rats could be a result of increased conversion of bigET-1 to active ET-1, or due to greater vascular smooth muscle sensitivity to ET-1. Since no differences were evident in ET-1 sensitivity/E_max_ between young and aged non-pregnant and pregnant rats (Figures 6C,D and Supplementary Table 7), it appears that there was a greater capacity for aged vessels to convert bigET-1 to ET-1. To determine which enzymes may be contributing to this enhanced conversion, inhibitors of various bigET-1 conversion enzymes were used. Incubating the vessels with the MMP-inhibitor GM6001 did not alter vasoconstriction responses to bigET-1 in either young or aged non-pregnant and pregnant rats (Figures 7A–D and Supplementary Table 8). We further evaluated the contribution of other enzymes involved in converting bigET-1 into its vasoactive form. Pre-incubation with the ECE-1 inhibitor CGS35066 did not impact bigET-1 responses in young non-pregnant rats but decreased maximum constriction (E_max_) was observed in pregnant rats (Figures 8A,B and Supplementary Table 8), while constriction to bigET-1 was significantly reduced in the aged non-pregnant and pregnant rats (Figures 8C,D). However, Western blot analysis showed that there were no changes in ECE-1 protein levels between the groups (Figure 8E). Chymase inhibitor chymostatin and neutral endopeptidase inhibitor thiorphan did not alter bigET-1 vasoconstriction in either young or aged rats (Supplementary Table 9).

In addition, no changes in the vasoconstriction capacity to KPSS were observed between the young (non-pregnant: 5.17 ± 0.41; *n* = 16 vs. pregnant: 5.51 ± 0.48 mN/mm; *n* = 12) and aged rats (non-pregnant; 5.85 ± 0.32; *n* = 15 vs. pregnant: 5.68 ± 0.38 mN/mm; *n* = 15), suggesting that overall constrictor capacity of the vascular smooth muscle cells was not different between groups.

## Discussion

The goal of the current study was to understand the vascular pathways that may be contributing to impaired vascular adaptations to pregnancy in a rat model of advanced maternal age. Overall, our data demonstrated altered vascular function in the aged non-pregnant rats, while these changes appear to be compensated for during pregnancy with a major reduction in blood pressure accompanied by an increased contribution of NO and EDH-mediated relaxation in aged pregnant rats. Furthermore, we demonstrated that contractile responses to bigET-1 were greater in mesenteric arteries of the aged groups compared with the young groups, independent of pregnancy. In contrast to our hypothesis, no contribution of MMPs in converting bigET-1 to active ET-1 was observed, while an increased contribution with ECE-1 was found in aged rats (both non-pregnant and pregnant). However, a similar contractile response to ET-1 suggests that the vascular smooth muscle sensitivity to ET-1 was not different between the groups. We speculate that, although aging contributes to changes in various pathways of endothelium-dependent vasodilation, pregnancy in aged dams may confer vascular protection.

### Advanced Maternal Age and Blood Pressure

It has been well established that aging alters the structural and functional properties of the vascular wall, leading to arterial stiffness with increased blood pressure, resulting in an overall constrictive phenotype (Boss and Seegmiller, 1981; Franklin, 1999; Lee and Oh, 2010; Toda, 2012). In the current study, the significantly higher blood pressure (systolic, diastolic, and mean arterial pressure) in non-pregnant aged rats compared to the other groups was not surprising, considering the prevalence of age-related arterial stiffness and hypertension (Lee and Oh, 2010; Xu et al., 2017). Of note, the systolic blood pressure appears to be a better predictor for cardiovascular disease with advancing age due to an increasing stiffness of large arteries (Franklin, 1999). Moreover, an elevated diastolic blood pressure typically correlates with impaired left ventricular relaxation, while mean arterial pressure in the normal range allows for adequate perfusion of vital organs (Benetos et al., 1997; Domanski et al., 1999; Franklin, 1999). Thus, in our aged non-pregnant rats, elevated blood pressures may be reflective of inherent cardiovascular changes associated with aging. Generally, a mild decrease in blood pressure is observed over the course of a normal pregnancy, being the lowest around mid-gestation, likely reflecting a reduced systemic vascular resistance (Villar and Sibai, 1989). We did not observe a difference in blood pressure between young non-pregnant and pregnant rats, which may, in part, be due to the fact that blood pressure was assessed in late gestation.

Interestingly, a significant reduction in blood pressure was observed in aged pregnant rats compared to the aged non-pregnant rats, such that young and aged pregnant rats had similar blood pressures. In aged pregnancies, this reduction in blood pressure may be due to an increased contribution of endothelium-dependent vasodilator pathways that we observed in aged pregnant rats (i.e., NO and EDH, discussed below). In line with our observations, Gaillard et al. (2011) in a population-based prospective cohort study, showed that blood pressure differences appear to be small and within the normal physiological range between young and older women during pregnancy. In contrast, Plant et al. (2020) showed an increased in diastolic blood pressure, but no changes in the systolic blood pressure in pregnant vervet/African green monkeys of advanced maternal age (3–9 years were considered optimal maternal age, while those 10 years and older were considered to be advanced maternal age). In addition, a trend in increase heart rate was observed in young pregnant rats compare to non-pregnant rats, consistent with the well-characterized cardiovascular adaptation to pregnancy seen in women (May, 2015). Interestingly, this trend was not observed between the aged non-pregnant and pregnant rats. This difference may be due to an impaired cardiac sympathetic activity in aging or may be related to cardiac remodeling that is known to occur in aging (Ferrari et al., 2003; Strait and Lakatta, 2012; Jakovljevic, 2018). While examining the adaptations to cardiac structure and function in our aged rat model was beyond the scope of the current study, future experiments will be designed to further investigate these findings. Overall, our study is unique in that we analyzed blood pressure changes compared to the non-pregnant state thus allowing us to assess the cardiovascular adaptations to pregnancy and with aging. As blood pressure was elevated in the aged non-pregnant rats, these animals may be entering pregnancy with a compromised cardiovascular capacity; and unless significant vascular adaptations to pregnancy occur, the aged cardiovascular system would likely be unable to support normal fetal growth and development.

### Advanced Maternal Age and Endothelium-Dependent Vasodilation

Normal pregnancy is associated with an increased endothelium-dependent vascular relaxation, with increased NO contribution during pregnancy (Morris et al., 1995; Williams et al., 1997). In the current study, although we did not observe any overall differences in endothelium-dependent vasodilation to MCh in mesenteric arteries between young and aged non-pregnant and pregnant rats, pre-treatment with the NOS inhibitor L-NAME revealed a significant decrease in E_max_ in both young and aged pregnant rats. These data suggest that in our model, NO contribution to MCh-induced vasodilation was increased during pregnancy, independent of aging. Interestingly, this greater NO contribution in pregnancy may be due to increased eNOS phosphorylation at Ser1177/activity, which was increased in aged pregnant rats (and tended to be higher in young pregnant rats) compared to both non-pregnant rat groups. Several studies clearly demonstrated increased contribution of NO in young pregnant animals in different vascular beds including mesenteric arteries (Conrad et al., 1993; Pascoal et al., 1995; Ni et al., 1997; Sladek et al., 1997; Choi et al., 2002; Cooke and Davidge, 2003; Hodzic et al., 2017; Owusu Darkwa et al., 2018), but there is paucity of research investigating systemic vascular contribution of NO in advanced maternal age. In non-pregnant animals, the shift in MCh-induced vasodilation by L-NAME in young but not aged rats suggests a reduced NO contribution in aged non-pregnant rats. These data support evidence in the literature that the NO pathway is important in vascular adaptations to pregnancy (Boeldt et al., 2011; Boeldt and Bird, 2017), including in our rat model of advanced maternal age.

In addition to NO, we also observed an EDH contribution to vasodilation (as assessed by inhibiting SK and IK channels with apamin and TRAM-34) in mesenteric arteries of young non-pregnant, aged non-pregnant and aged pregnant rats, however, this contribution of EDH was absent in young pregnant rats. Thus, in young pregnant rats, the EDH contribution to vasodilation (specifically via SK and IK channels) seems minimal, while this pathway may be maintained in aged pregnant rats as an additional endothelium-dependent mechanism that has adapted with aging to compensate for the enhanced constrictor state, thus supporting pregnancy. A variety of other EDH-pathways contribute to vasodilation, such as epoxyeicosatrienoic acids (EETs), hydrogen peroxide, and MEGJs (Chen et al., 1988; Corriu et al., 1998; Vanhoutte, 2004; Ng et al., 2008). We did not observe any contribution of MEGJs to MCh-induced vasodilation. Although assessing all other EDH pathways was beyond the scope of the current study, and it will be interesting to evaluate these specific pathways in future experiments. In contrast to our findings, others have shown a loss of EDH-mediated vasodilation in rat mesenteric arteries with aging and hypertension, in part due to decrease synthesis/release of EDH or a defective electrical coupling between endothelial and smooth muscle cells (Fujii et al., 1993; Goto et al., 2004). Furthermore, in normal pregnancy, EDH-mediated vasodilation was elevated due to increased synthesis/reduced degradation in addition to NO/prostanoid synthesis, contributing to the vascular adaptations (Gerber et al., 1998). Thus, EDH contribution to vasodilation exists based on the species, nature (young vs. aged), vascular bed, and state (pregnant vs. non-pregnant), and the enhanced EDH-dependent relaxation in conditions such as vascular aging may be a compensatory mechanism to maintain a balance of vasoactive factors. Moreover, the overall contribution of NO in young pregnant rats and increased NO and EDH contribution in aged pregnant rats did not account for all of the MCh-induced vasodilation. As such, there might be a significant contribution of other vasodilator pathways, such as prostacyclin, contributing to endothelium-dependent vasodilation, would be interesting to assess in future studies. Overall, our data indicate that NO is involved in endothelium-dependent vasodilation in both young and aged pregnant rats, while enhanced EDH-mediated vasodilation in mesenteric arteries from aged pregnant rats (and a decrease in blood pressure) suggests that beneficial vascular adaptations occur in the aged rats that were able to maintain pregnancy.

### Advanced Maternal Age and Vasoconstriction

The ET-1 pathway plays an essential role in the maintenance of vascular tone, however, a greater ET-1 activity is associated with pathological conditions, such as aging, and may impair vascular function (Westby et al., 2011; Wang et al., 2014; Freitas-Rodriguez et al., 2017; Xu et al., 2017). ET-1 production involves enzymatic cleavage of bigET-1. In the current study, constriction responses to bigET-1 were increased in the aged groups, and since the vascular smooth muscle response to ET-1, as well as to KPSS (suggesting no change in non-receptor mediated vasoconstriction responses) was not different among the groups, this enhanced bigET-1 responsiveness appeared to be due to increased bigET-1 processing in aged arteries. We postulated this may be due to upregulation of MMPs in the aged animals. However, we did not observe a significant contribution of MMPs-mediated constriction to bigET-1 in mesenteric arteries from any of our group. It is possible that our aged rat model (9.5 months ∼35 years of human age) is in fact too “young” and do not yet demonstrate an aged-associated increase in MMP expression/activity, which has been described by others at older ages (Wang and Lakatta, 2002; Lindsey et al., 2005; Lekontseva et al., 2010; Wang et al., 2015; Freitas-Rodriguez et al., 2017). Moreover, MMP-contribution may be vascular bed-dependent, as suggested by other studies (Crowther et al., 2000; Wang et al., 2012, 2014). Thus, in the mesenteric arteries of our aged rats, MMPs may not play a significant role, while other enzymes may contribute to bigET-1 conversion, such as ECE-1.

ECE-1 has been shown to play a dominant role in the processing of bigET-1 in aging vasculature (Stauffer et al., 2008; Goel et al., 2010). Indeed, we showed a significant ECE-1 contribution to bigET-1 cleavage in aged mesenteric arteries, independent of pregnancy state, while in young rats there was no role for ECE-1 conversion of bigET-1. Interestingly, we did not observe any changes in the ECE-1 expression between the groups, thus the increased ECE-1 contribution may be due to an enhanced ECE-1 activity rather than expression. Although the cleavage of bigET-1 to active ET-1 is primarily through MMPs and ECE-1, alternative pathways including chymase and neutral endopeptidases are also involved (De Campo et al., 2002; Fecteau et al., 2005; Simard et al., 2009). However, our data did not support a major role for these enzymes. Nevertheless, a constitutive conversion of bigET-1 to the potent vasoconstrictor ET-1 occurs by several enzymes to maintain normal vascular tone, and the to best of our knowledge, we are the first to demonstrate the enhanced contribution of ECE-1 in converting bigET-1 to ET-1 in a rat model of advanced maternal age. Our findings of increased NO and EDH contribution to vasodilation, together with enhanced ECE-1-mediated conversion of bigET-1 to ET-1 in mesenteric arteries, suggests an important link between endothelium-derived NO and EDH signaling and the bigET-1/ET-1 pathways. Indeed, future studies are warranted to determine how these mechanisms are interacting and contributing to systemic vascular adaptations to pregnancy a rat model of advanced maternal age.

## Conclusion

The population of women becoming pregnant at an advanced age is increasing globally and poses many health challenges due to their increased risk of pregnancy complications. As such, women of advanced age represent a very important and yet understudied demographic of pregnant women. Our study explores various vascular pathways involved in the adaptations to pregnancy, which were distinct in advanced maternal age compared to young rats. Although NO is involved in the vasodilation in both young and aged pregnant rats, enhanced EDH mediated endothelium-dependent vasodilation in aged mesenteric vasculature and lower MAP suggests a beneficial adaptation in these rats that were able to maintain pregnancy. In addition, increased contribution of ECE-1 may provide a more dominant conversion pathway for bigET-1 in aging vasculature. This study increases our understanding of the vascular pathways involved in the systemic vascular adaptations to pregnancy. However, maternal aging is often associated with co-morbidities, thus future studies including a “second hit” in our animal model may provide more insight. For example, exposing rats to a high fat diet (obesity) or high salt (hypertension) may unmask underlying vascular deficits in the current model of advanced maternal age, and may thus provide additional clinically relevant insights. Given the increasing trend toward delaying pregnancy, understanding the vascular adaptations that may be compromised, thus contributing to an increased risk of adverse pregnancy outcomes in women with advanced maternal age, may help to develop effective treatment and prevention strategies.

## Data Availability Statement

The raw data supporting the conclusions of this article will be made available by the authors, without undue reservation.

## Ethics Statement

The animal study was reviewed and approved by the University of Alberta Health Sciences Animal Policy and Welfare Committee.

## Author Contributions

MP, SD, FS, and C-LC: study conception and design, analysis, and interpretation of data. MP, AW, and RK: acquisition of data. MP: drafting of the manuscript. MP, AW, FS, SD, and C-LC: critical revision of the manuscript. All authors contributed to the article and approved the submitted version.

## Conflict of Interest

The authors declare that the research was conducted in the absence of any commercial or financial relationships that could be construed as a potential conflict of interest.

## Publisher’s Note

All claims expressed in this article are solely those of the authors and do not necessarily represent those of their affiliated organizations, or those of the publisher, the editors and the reviewers. Any product that may be evaluated in this article, or claim that may be made by its manufacturer, is not guaranteed or endorsed by the publisher.
