# Supplementary material for: Altered Vascular Adaptations to Pregnancy in a Rat Model of Advanced Maternal Age
Source: Front Physiol. 2021 Jul 28;12:718568. doi: 10.3389/fphys.2021.718568 (PMC8356803; doi:10.3389/fphys.2021.718568)
Supplement: Supplementary file 1 [file Data_Sheet_1.pdf]

Supplementary Data

	Young		Aged	
	Non-pregnant	Pregnant	Non-pregnant	Pregnant
Diastolic pressure (mmHg)	90.1 ± 3.71	84.7 ± 1.87	108.7 ± 2.35***	89.3 ± 2.95
Systolic pressure (mmHg)	120.3 ± 3.21	113.1 ± 3.38	147.0 ± 4.19***	120.6 ± 3.06
Heart Rate (BPM)	375.2 ± 12.6	419.4 ± 15.0#	390.3 ± 13.49	404.3 ± 13.35

Supplementary Table 1. Systolic, diastolic blood pressure (mmHg) and heart rate (BPM) readings from young and aged non-pregnant and pregnant rats on gestational day 20. Data presented as mean±SEM; analyzed by two-way ANOVA with Sidak's multiple comparisons post-hoc test; #p=0.058; ****p<0.0001 compared to aged pregnant, young non-pregnant and pregnant rats; n= 9-10/group.

	Young		Aged	
	Non-pregnant	Pregnant	Non-pregnant	Pregnant
E_{max} (% vasodilation)	98.83 ± 4.81	99.21± 1.00	97.82 ± 3.42	99.29 ± 1.54
AUC (arbitrary units)	330.64 ± 11.66	345.15 ± 13.68	323.49 ± 9.16	351.95 ± 9.66

Supplementary Table 2. Endothelium-dependent vasodilation responses to methacholine (MCh) in mesenteric arteries from young and aged non-pregnant and pregnant rats. Summarized as the maximal vasodilation responses (E_{max}; % vasodilation) and area under the curve (AUC; arbitrary units) to MCh. Data presented as mean±SEM; analyzed by two-way ANOVA with Sidak's multiple comparisons post-hoc test; n=6-11/group.

	Young		Aged	
	Non-pregnant	Pregnant	Non-pregnant	Pregnant
Control (AUC)	330.64 ± 11.66	345.15 ± 13.68	323.49 ± 9.16	351.95 ± 9.66
L-NAME (AUC)	263.40 ± 13.96**	272. 94 ± 24.92*	291.76 ± 8.56	265.11 ± 21.35**

Supplementary Table 3. Contribution of the nitric oxide (NO) to endothelium-dependent vasodilation responses to methylcholine (MCh) in mesenteric arteries from young and aged non-pregnant and pregnant rats. Summarized as the area under the curve (AUC; arbitrary units) to MCh after pre-incubation with the NO-inhibitor L-NAME or without inhibitor (control). Data presented as mean±SEM; analyzed by two-way ANOVA with Sidak's multiple comparisons post-hoc test; *p<0.05, **p<0.01 – L-NAME versus Control; n=6-11/group.

	Young		Aged	
	Non-pregnant	Pregnant	Non-pregnant	Pregnant
Control (AUC)	327.91 ± 11.81	349.37 ± 25.09	366.27 ± 13.56	359.38 ± 19.46
Apamin/TRAM-34 (AUC)	266.68 ± 26.84	351.28 ± 28.86	255.10 ± 24.63*	274.63 ± 34.65*

Supplementary Table 4. Contribution of the EDH to endothelium-dependent vasodilation responses to methylcholine (MCh) in mesenteric arteries from young and aged non-pregnant and pregnant rats. Summarized as the area under the curve (AUC; arbitrary units) to MCh after pre-incubation with EDH inhibitor - apamin/TRAM-34 or without inhibitor (control). Data presented as mean±SEM; analyzed by two-way ANOVA with Sidak's multiple comparisons post-hoc test; *p<0.05 – Apamin/TRAM-34 versus Control; n=7-8/group.

	Young		Aged	
	Non-pregnant	Pregnant	Non-pregnant	Pregnant
Control (pEC_{50})	7.26 ± 0.31	7.41 ± 0.35	7.50 ± 0.22	7.55 ± 0.42
18α-glycyrrhetinic acid (pEC_{50})	7.36 ± 0.42	7.73 ±0.15	7.43 ± 0.49	7.76 ± 0.42
Control (E_{max})	97.50 ± 1.39	101.15 ± 2.97	99.94 ± 0.18	97.91 ± 1.19
18α-glycyrrhetinic acid (E_{max})	96.42 ± 1.12	98.75 ± 0.45	98.44 ± 0.45	98.30 ± 0.51
Control (AUC)	315.41 ± 19.65	349.37 ± 25.09	366.27 ± 13.56	359.38 ± 19.46
18α-glycyrrhetinic acid (AUC)	330.19 ± 23.46	371. 21 ± 24.04	363.41 ± 18.08	377.95 ± 16.20

Supplementary Table 5. Contribution of the myoendothelial gap junctions (MEGJs) to endothelium-dependent vasodilation responses to methylcholine (MCh) in mesenteric arteries from young and aged non-pregnant and pregnant rats. Summary of the sensitivity to MCh (pEC_{50}), maximal vasodilation responses (E_{max}; % vasodilation) and area under the curve (AUC; arbitrary units) to MCh after pre-incubation with the MEGJ-inhibitor 18a-glycyrrhetinic acid or without inhibitor (control). Data presented as mean±SEM; analyzed by two-way ANOVA with Sidak's multiple comparisons post-hoc test; n=5-8/group.

	Young		Aged	
	Non-pregnant	Pregnant	Non-pregnant	Pregnant
SNP E_{max} (% vasodilation)	96.68 ± 1.59	93.32 ± 8.80	93.93 ± 7.59	95.59 ± 3.00
SNP pEC50	7.55 ± 0.08	7.85 ± 0.16	7.43 ± 0.10	7.65 ± 0.10
SNP (AUC)	315.41 ± 19.65	300.68 ± 10.74	242.75 ± 7.32	271.86 ± 11.68

Supplementary Table 6. Endothelium-independent vasodilation responses to sodium nitroprusside (SNP) in mesenteric arteries from young and aged non-pregnant and pregnant rats. Summarized as the maximal vasodilation responses (E_{max}; % vasodilation), sensitivity to SNP (pEC_{50}), and area under the curve (AUC; arbitrary units) to SNP. Data presented as mean±SEM; analyzed by two-way ANOVA with Sidak's multiple comparisons post-hoc test; n=5-8/group.

	Young		Aged	
	Non-pregnant	Pregnant	Non-pregnant	Pregnant
Big-ET-1 (E_{max}; mN/mm)	4.56 ± 0.22	4.95 ± 0.27	6.56 ± 0.50	6.14 ± 0.64
ET-1 (E_{max}; mN/mm)	5.37 ± 0.53	5.95 ± 0.37	7.10 ± 0.68	6.42 ± 0.65

Supplementary Table 7. Vascular responses to bigET-1 and ET-1 in mesenteric arteries from young and aged non-pregnant and pregnant rats. Summarized as the maximal vasocontraction responses (Emax; mN/mm) to bigET-1 and ET-1. Data presented as mean±SEM; analyzed by two-way ANOVA with Sidak's multiple comparisons post-hoc test; n=6-16/group.

	Young		Aged	
	Non-pregnant	Pregnant	Non-pregnant	Pregnant
Control (E_{max})	4.56 ± 0.22	4.95 ± 0.27	6.56 ± 0.50	6.14 ± 0.64
GM6001 (E_{max})	5.28 ± 0.32	3.99 ± 0.42	5.38 ± 0.62	4.71 ± 0.55
CGS35066 (E_{max})	3.96 ± 0.12	3.49 ± 0.15*	3.46 ± 0.49**	2.92 ± 0.27**

Supplementary Table 8. Contribution of matrix metalloproteases (MMPs) and endothelin converting enzymes (ECE-1) to the conversion of bigET-1 to ET-1 in mesenteric arteries from young and aged non-pregnant and pregnant rats. Summarized as the maximal vasocontraction responses (Emax; mN/mm) to bigET-1 responses after pre-incubation with GM6001 (MMPs inhibitor) or CGS35066 (ECE-1 inhibitor), or without inhibitor (control). Data presented as mean±SEM; analyzed by two-way ANOVA with Sidak's multiple comparisons post-hoc test; *$p<0.05$, **$p<0.01$– CGS35066 versus Control; n=6-16/ group.

	Young		Aged	
	Non-pregnant	Pregnant	Non-pregnant	Pregnant
Control (AUC)	0.49 ± 0.06	0.77 ± 0.18	1.09 ± 0.22	0.92 ± 0.13
Chymostatin (AUC)	0.45 ± 0.10	0.48 ± 0.15	0.74 ± 0.18	0.73 ± 0.16
Thiorphan (AUC)	0.78 ± 0.14	0.82 ± 0.20	1.29 ± 0.23	0.96 ± 0.20
Control (E_{max})	3.13 ± 0.27	4.23 ± 0.47	6.78 ± 0.76	7.79 ± 1.13
Chymostatin (E_{max})	3.21 ± 0.53	2.98 ± 0.54	6.11 ± 1.23	6.12 ± 1.03
Thiorphan (E_{max})	3.87 ± 0.24	3.41 ± 0.33	6.42 ± 0.73	6.32 ± 0.83

Supplementary Table 9. Contribution of chymases and neutral endopeptidases to the conversion of bigET-1 to ET-1 in mesenteric arteries from young and aged non-pregnant and pregnant rats. Summarized as the area under the curve (AUC; arbitrary units) and maximal vasocontraction responses (Emax; mN/mm) to bigET-1 responses after pre-incubation with chymostatin (chymase inhibitor) or thiorphan (neutral endopeptidase inhibitor), or without inhibitor (control). Data presented as mean±SEM; analyzed by two-way ANOVA with Sidak's multiple comparisons post-hoc test; n=6-8/ group.

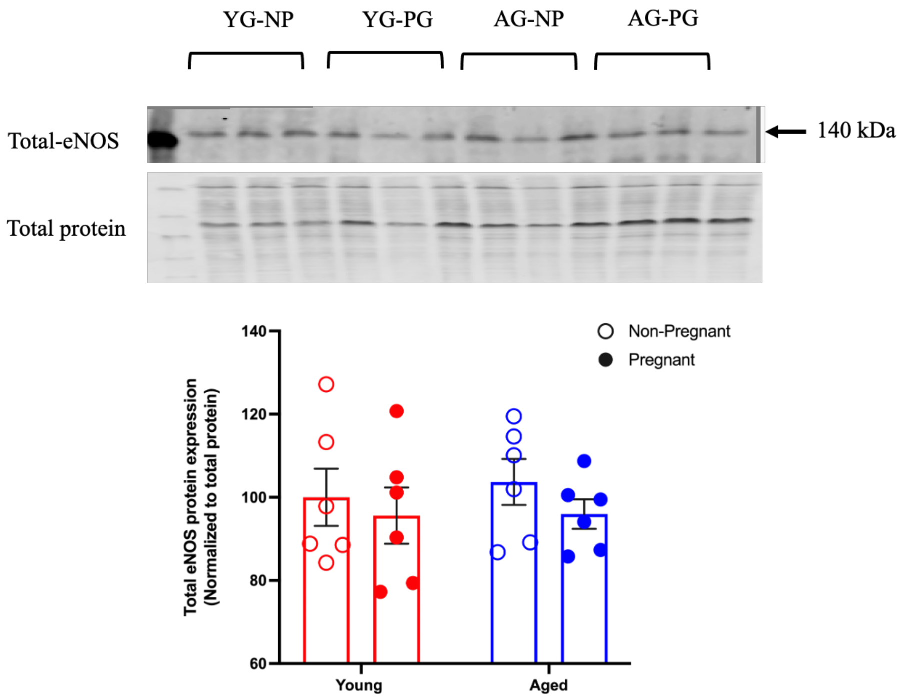

Supplementary Figure 1. Total eNOS protein expression in mesenteric arteries of young and aged pregnant rats. Western blot analysis of total eNOS protein expression, normalized to total protein, in mesenteric arteries of young (3-4 months; in red) and aged (9-9.5 months; in blue) pregnant (on gestational day 20; closed circles) and non-pregnant (age-matched; open circles) rats. Data are presented as mean±SEM and expressed as percentage of control (i.e. the mean of the non-pregnant young group); analyzed by two-way ANOVA with Sidak's multiple comparisons post-hoc test; n=6/group. YG-NP=Young non-pregnant; YG-PG=Young pregnant; AG-NP=Aged non-pregnant; AG-PG= Aged pregnant.
